# Is Non-Homologous End-Joining Really an Inherently Error-Prone Process?

**DOI:** 10.1371/journal.pgen.1004086

**Published:** 2014-01-16

**Authors:** Mireille Bétermier, Pascale Bertrand, Bernard S. Lopez

**Affiliations:** 1 CNRS, Centre de Génétique Moléculaire, UPR3404, Gif-sur-Yvette, France; 2 CNRS, Centre de Recherches de Gif-sur-Yvette, FRC3115, Gif-sur-Yvette, France; 3 Université Paris-Sud, Département de Biologie, Orsay, France; 4 CEA, DSV, Institut de Radiobiologie Moléculaire et Cellulaire, Laboratoire Réparation et Vieillissement, Fontenay-aux-Roses, France; 5 UMR 8200 CNRS, Villejuif, France; 6 Institut de Cancérologie, Gustave Roussy, Villejuif, France; Duke University, United States of America

## Abstract

DNA double-strand breaks (DSBs) are harmful lesions leading to genomic instability or diversity. Non-homologous end-joining (NHEJ) is a prominent DSB repair pathway, which has long been considered to be error-prone. However, recent data have pointed to the intrinsic precision of NHEJ. Three reasons can account for the apparent fallibility of NHEJ: 1) the existence of a highly error-prone alternative end-joining process; 2) the adaptability of canonical C-NHEJ (Ku- and Xrcc4/ligase IV–dependent) to imperfect complementary ends; and 3) the requirement to first process chemically incompatible DNA ends that cannot be ligated directly. Thus, C-NHEJ is conservative but adaptable, and the accuracy of the repair is dictated by the structure of the DNA ends rather than by the C-NHEJ machinery. We present data from different organisms that describe the conservative/versatile properties of C-NHEJ. The advantages of the adaptability/versatility of C-NHEJ are discussed for the development of the immune repertoire and the resistance to ionizing radiation, especially at low doses, and for targeted genome manipulation.

DNA double-strand breaks (DSBs) are highly toxic lesions. However, in certain essential physiological processes, DSBs are used to promote genetic diversity. Programmed DSBs generated by cellular enzymes are repaired by the same mechanisms as those used for stress-induced DSBs. Thus, DSB repair stands at the crossroads between genetic variability and instability.

DSB repair uses two primary strategies: non-homologous end-joining (NHEJ), which is generally considered to be error-prone, and homologous recombination (HR), which is considered to be error-free. However, this view is too simplistic. Herein, we discuss several pieces of data that challenge the fallibility of NHEJ.

## Canonical NHEJ *versus* Alternative End-Joining

**Figure 1:**
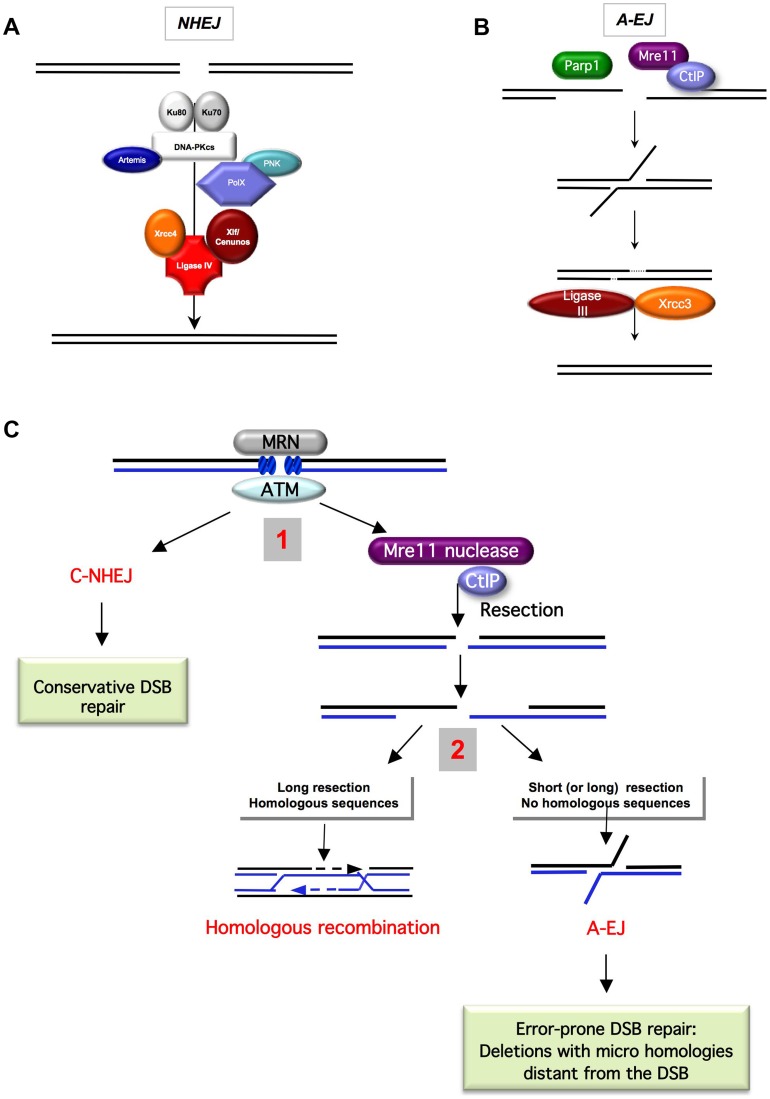
End-joining models and competition between C-NHEJ and A-EJ for DSB repair. **A**) **The canonical C-NHEJ.** The heterodimer Ku80-Ku70 binds to the DNA ends, which then recruit DNA-PKcs. Note that DNA-PK is absent from yeast. Several proteins, including Artemis, the polynucleotide kinase (PNK), and members of the polymerase X family, process the DNA ends for subsequent steps [86]–[90]. In the last step, ligase IV, associated with its co-factors Xrcc4 and Cernunos/Xlf, joins the ends [91]–[93]. **B**) **A-EJ.** Parp1 plays a role in the initiation process [4], [17], [94], [95]. Without the protection by Ku70/Ku80, the DNA ends are resected in a reaction favored by the nuclease activity of Mre11 and CtIP [11], [13]. It has been proposed that a single-strand DNA resection reveals complementary microhomologies (two to four nt or more) that can anneal; gap filling completes the end-joining. Subsequently, Xrcc1 and ligase III (which can be substituted by ligase I) complete A-EJ [4], [9], [38]. A-EJ is always associated with deletions at the junctions and frequently (but not systematically) involves microhomologies that are distant from the DSB. The histone H1 has also been shown to act in A-EJ [96]. **C**) **Two-step model for the choice of the DSB repair pathway** [**3**], [**11**] **.** The MRN complex and ATM are involved in the early steps of DSB signaling and can activate both C-NHEJ and A-EJ. **1**) Binding of Ku80/Ku70 protects from ssDNA resection, leading to a conservative DSB repair outcome through C-NHEJ. The nuclease activity of Mre11 and CtIP can initiate ssDNA resection. **2**) A short ssDNA resection allows A-EJ but not homologous recombination. A long ssDNA resection allows A-EJ and HR, but HR requires the presence of homologous sequences. A-EJ results in error-prone repair associated with deletions at the repair junctions with frequent use of microhomologies distant from the DSB.

The canonical C-NHEJ pathway joins double-strand DNA ends in a Ku- and Xrcc4/ligase IV–dependent manner. This pathway has been extensively described and is summarized in Figure 1A.

The existence of alternative end-joining pathways has been recently reported (Figure 1B). This alternative end-joining process, which can be unmasked in the absence of functional C-NHEJ genes, is referred to as A-EJ or alt-NHEJ (alternative end-joining), B-NHEJ (backup NHEJ), and MMEJ (microhomology-mediated end-joining) [1]–[11]. Herein, to clearly distinguish it from C-NHEJ and because some repair events do not use microhomologies, it will be referred to as A-EJ. A-EJ is far from being fully characterized and might correspond to different molecular processes [12], but the common points are that it does not require extended sequence homologies, is independent of Ku80 or Xrcc4, and is associated with deletions at the repair junctions, frequently (but not systematically) using microhomologies distant from the DSB. This signature led to the model in Figure 1B, which proposes that A-EJ is initiated by a single-stranded (ssDNA) resection. Consistent with this view are the involvement of the nuclease activities of MRE11 and CtIP/Sae2 [11], [13], [14] and the fact that 53BP1, in association with RIF1 and BLM, protects against long deletions at the A-EJ repair junctions [15]. Consequently, A-EJ is highly mutagenic, typically generating deletions at the repair junction. Because HR is also initiated by a ssDNA resection, a two-step model has been proposed for the choice of the DSB repair pathway [3], [11]. The first alternative is the choice between C-NHEJ and the initiation of the resection; the second alternative is HR *versus* A-EJ (Figure 1C). Consistent with the first alternative, Ku represses both HR and A-EJ [1], [2], [7], [8], [16], [17]. A defect in Ku leads to extended DNA degradation at the DSBs and to increased deletion sizes at the A-EJ junctions [2], [6], [18]–[20]. Note that a defect in Ku does not significantly decrease, whereas the absence of Xrcc4 leads to a strong decrease in the total efficiency of end-joining [1], [21]. In both cases, the remaining events exhibit the signature of A-EJ at the repair junction (deletions). This shows that the absence of Ku is compensated by A-EJ. In the absence of Xrcc4, Ku is still present and able to repress A-EJ, thus independently of the late steps of C-NHEJ. These data support the concept that Ku protects against initiation of A-EJ. Because A-EJ is exclusively mutagenic, Ku favors the maintenance of genetic stability.

Several parameters affect the second choice, such as the presence of a homologous sequence. Moreover, long resections are required for HR (hundreds of nucleotides), whereas short resections (a few tens of nucleotides) are sufficient for A-EJ, as estimated by the deletion sizes at the repair junctions. Nevertheless, long deletions can also lead to A-EJ. The cell cycle can also affect the DSB repair pathway choice; HR is only active in the S and G2 phases [22]–[26], whereas both C-NHEJ and A-EJ are active throughout the cell cycle [23], [24], but A-EJ is more active in the S phase [23].

## C-NHEJ Is a Conservative but Versatile DSB Repair Process

Genetic instability can be evaluated at two levels: at the chromosome level or at the nucleotide level, at the DSB repair scar.

### At the Chromosome Level

C-NHEJ can be involved in translocations and rearrangements [2], [27] and in programmed rearrangements (generating the immune repertoire). Whole genome sequencing of tumors has revealed complex inter- and intra-chromosomal rearrangements in a phenomenon named chromothripsis. Both C-NHEJ and A-EJ have been proposed to be involved in chromothripsis, but they cannot account for events involving sequence duplication (for review see [28], [29]).

Nevertheless, a defect in Ku or Xrcc4/lig IV leads to profound genome rearrangements, underlying the fact that NHEJ is essential for the maintenance of genomic stability [10], [30]–[33]. Additionally, NHEJ prevents trinucleotide repeat fragility and expansion [34]. Conversely, A-EJ is involved in chromosome translocation in mouse cells, *Drosophila*, and yeast cells [35]–[37]. Particularly, both CtIP and ligase III have been shown to be involved in translocations by A-EJ [38], [39].

The mobility of the DNA ends is a prerequisite to generate profound genome rearrangements. Remarkably, Ku80 protects broken DNA ends against mobility within the nucleus [40]. In addition, atomic force and electron microscopy studies have shown that Ku, in conjunction with DNA-PKcs, tethers DNA ends *in vitro* [41], [42], maintaining them in close proximity. Ku-mediated tethering could account for the protective role of Ku80 against the mobility of DNA ends and consequently against translocations. In addition, increased mobility of DSBs has been associated with DNA end resection in yeast, thus favoring the search for homology during HR [43], [44]. Because A-EJ is also initiated by DNA end resection, DSB mobility might also increase the risk of chromosome rearrangements promoted by A-EJ. Because Ku impairs both DSB mobility and DNA end resection, it likely plays a doubly protective role against chromosome rearrangements.

### At the Nucleotide Level, at the Repair Scar

At the repair junctions, the apparent infidelity of end-joining should be reevaluated because, in many studies, A-EJ was not distinguishable from C-NHEJ. In addition, the repair of DSBs induced by ionizing radiation (IR) or V(D)J recombination requires processing of the DNA ends prior to ligation. Thus, it can be argued that mutagenesis is generated by DNA end processing rather than by the end-joining machinery *per se*. Thus using biological systems that do not require DNA end processing is necessary to address the question of the actual accuracy of C-NHEJ.

#### The end-joining accuracy of directly ligatable DNA ends

We will first discuss NHEJ in two biological models, *Paramecium* and mammalian cells, in which this pathway is of particular importance. *Paramecium* provides a physiological example of the efficient contribution of C-NHEJ to the precise repair of thousands of developmentally programmed DSBs [45]. In mammalian cells, C-NHEJ is a prominent DSB repair mechanism, and it is essential in fundamental processes establishing the immune repertoire. The accuracy of NHEJ will then be addressed in yeast, bacteria, and plants, and during cut-and-paste transposition.

**Figure 2:**
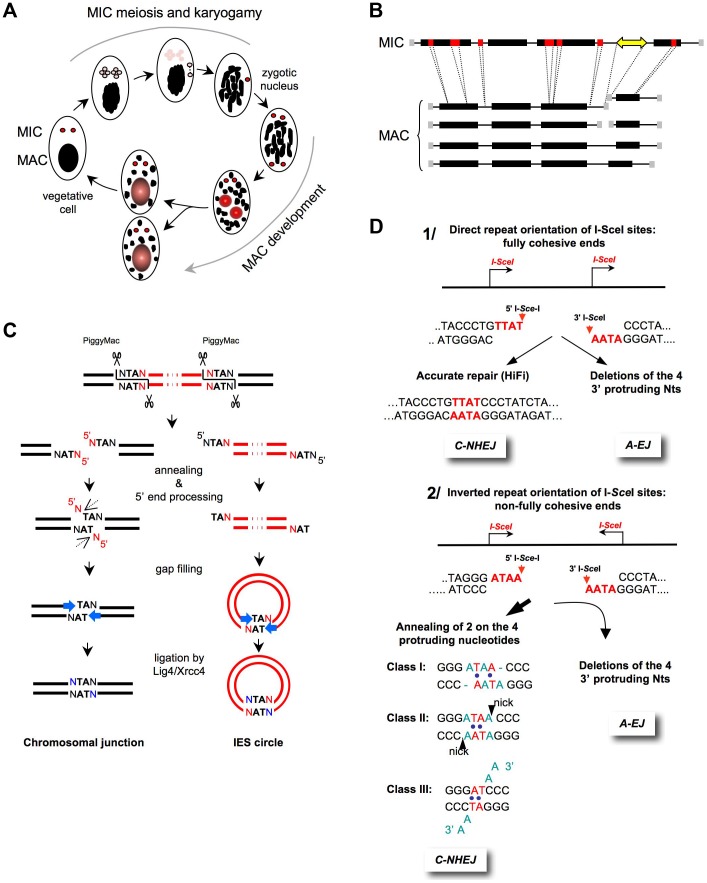
End-joining accuracy of ligation-compatible ends. **A**) **The** ***Paramecium*** **sexual cycle.** Two types of sexual processes are induced through starvation in *Paramecium*: autogamy, a self-fertilization process (shown in the figure), and conjugation between compatible mating types (not shown). During autogamy, the two germline diploid MICs (red) undergo meiosis to generate eight haploid nuclei (pink), and a single nucleus migrates to a specialized cell compartment, dividing once to produce two identical gametic nuclei. The remaining seven meiotic products are degraded, and the old MAC (black) becomes fragmented. During karyogamy, the two gametic nuclei fuse to form a diploid zygotic nucleus. The zygotic nucleus subsequently undergoes two successive mitotic divisions; after the second division, the two nuclei become the new MICs of the sexual progeny (red), whereas the other two differentiate into new developing MACs (red and gray) and undergo programmed genomic rearrangements. At the first cell division, the new MICs divide mitotically, and each of the two developing new MACs segregates into a daughter cell where it continues to amplify the rearranged genome to a final ploidy of ∼800 n. During conjugation, MIC meiosis is triggered through the mating of two compatible sexual partners, which undergo a reciprocal exchange of their haploid gametic nuclei. Consequently, the zygotic nucleus in each partner is formed through the fusion of a resident and a migratory haploid nucleus. Exconjugants separate between the first and second divisions of the zygotic nucleus, and MAC development occurs as described for autogamous cells. **B**) **General structure of MIC and MAC chromosomes.** On the MIC chromosomes, genes (black boxes) and non-coding regions (thin lines) are interrupted by short internal eliminated sequences (IESs in red). Repeated germline sequences (e.g., transposons and minisatellites) are indicated with a yellow double-headed arrow. During MAC development, each MIC chromosome is amplified ∼400-fold to generate a population of heterogeneous MAC chromosomes. The imprecise elimination of repeated DNA is associated with the following alternative rearrangements: i) chromosome fragmentation and telomere addition to new MAC chromosome ends (gray squares) and ii) imprecise joining of the two chromosome arms that flank the eliminated germline region. **C**) **Mechanism of IES excision.** The successive DNA intermediates formed during IES excision are displayed, with IESs shown in red and flanking MAC-destined DNA shown in black. The first step of the reaction is the introduction of 4-base staggered double-strand breaks at each IES end, depending upon the PiggyMac domesticated transposase. The molecular steps that lead to the repair of the chromosomal junction are shown on the left, which might occur within a paired-end intermediate through the annealing of the central TAs within each 5′ overhang. The removal of the 5′-terminal nucleotide was demonstrated *in vivo* (dotted arrow), but the nuclease(s) involved has not been identified. For the 3′-processing step, ligase IV (Lig4) recruits or activates a gap-filling DNA polymerase, which adds one nucleotide to the recessive 3′-end prior to the final ligation. A similar mechanism has been proposed for the circularization of excised linear IES molecules (right), provided that these molecules are sufficiently long. IES circles do not replicate and are actively degraded. **D**) **End-joining of fully** ***versus*** **non–fully complementary ends.** **1**) **I-** ***Sce*** **I sites in direct orientation (arrows).** The cleavage generates 3′-overhangs (red nt), which are fully complementary. C-NHEJ promotes accurate ligation (left panel), and A-EJ deletes the four protruding nucleotides, leading to the deletion of at least 4 bp at the resealed junction (right panel) [1], [2], [11]. **2**) **I-** ***Sce*** **I sites in an inverted orientation (arrows).** The cleavage generates 3′overhangs (red nt), which are not fully complementary. Similarly, A-EJ deletes the 3′-protruding nt, resulting in the deletion of at least 4 bp at the resealed junction (left panel). C-NHEJ anneals two of the four protruding nt (red nt), according to three classes of events (right panel). This imperfect annealing generates gaps (in blue in class I), mismatches (in blue in classes I and II), or 3′-single-stranded tails (in blue in class III) [1], [2], [11].

Similar to other ciliates, *Paramecium* harbors two different nuclei in its cytoplasm. During vegetative growth, the diploid micronucleus (MIC) divides through mitosis but remains transcriptionally silent, whereas the highly polyploid macronucleus (MAC) ensures gene expression. During sexual processes, the MAC is fragmented and eventually lost. Subsequent divisions of the zygotic nucleus produce the new MICs and MACs of the next sexual generation (Figure 2A). During the MAC development, the germline genome is amplified to a final ploidy of ∼800 n. Concomitantly, massive genome rearrangements occur [46]: i) repeated sequences, including transposons or minisatellites, are eliminated in a heterogeneous manner and ii) at least 45,000 short, non-coding intervening sequences, the IESs (Internal Eliminated Sequences), are excised [47] (Figure 2B). IESs excision generates one chromosomal DSB every 1–2 kb within a defined time window [48]. Thus, because endoduplication occurs during rearrangements, an estimated 10^6^ DSBs must be repaired in each developing MAC [49]. Despite this huge number, DSB repair preserves the linear organization of the MAC chromosomes. The highly precise repair of the IES excision sites occurs through the C-NHEJ pathway, as evidenced by the absolute requirement for ligase IV and Xrcc4 [50], but requires limited processing of DSBs (Figure 2C). Because 47% of the genes are interrupted by at least one IES in the MIC [47], the precision of end-joining is essential for the recovery of functional genes in the new MAC and, therefore, for cell survival.

In mammalian cells, different studies analyzing the end-joining of plasmids that are cleaved by restriction endonucleases in either acellular extracts or in transfected cells have all concluded that NHEJ is accurate [6], [51]–[53]. Defects in any C-NHEJ component resulted in error-prone end-joining [6], [52], [53], corroborating that mutagenic end-joining results, at least in part, from A-EJ. However, in these studies, the DSB repair was not monitored in a chromosomal context. Therefore, different systems, based on the use of intrachromosomal substrates containing cleavage sites for the meganuclease I-*Sce*I, have been studied. Notably, these experiments facilitated the characterization of A-EJ at a precise molecular level in the context of chromosomes in living cells [1], [2], [11], [21], [23], [54], [55]. The conclusions drawn from these studies (see below) were confirmed *in vivo* in mice in the context of physiological processes, such as class switch or V(D)J recombination [5], [10].

Because the I-*Sce*I cleavage site is not palindromic, the use of two *cis* sites (Figure 2D.1) has been informative [2]; with the sites in direct orientation, the I-*Sce*I–mediated cleavage generates two fully complementary ends that can be readily ligated, whereas in the inverted orientation, only partially complementary ends are generated (Figure 2B). Note that in these cases, the DNA ends are not chemically modified and, thus, are competent for the ligation machinery; the differences arise from the annealing of the four protruding nucleotides, which are fully complementary or not. In the latter case, end-joining cannot restore the initial sequence, generating an apparently mutagenic event. Notably, the efficiency of the joining of imperfectly complementary ends is similar to that of fully complementary ends, underlying the adaptation capabilities of NHEJ [2].

With fully complementary ends (I-*Sce*I sites in direct orientation), an error-free event restores one I-*Sce*I cleavage site. Thus, with these substrates, the frequency of error-free end-joining is underestimated because residual I-*Sce*I protein can re-cleave the repaired junction, increasing the possibility for error-prone repair and introducing a bias in favor of inaccurate repair. The frequency of error-free events in wild-type cells consistently varies from 35% to 75%, according to the level of I-*Sce*I expression and the half-life of the expressed I-*Sce*I protein [1], [2], [11]. Nevertheless, the high frequency of error-free events (up to 75%, which is likely underestimated) shows that C-NHEJ should not be primarily error-prone in mammalian cells. Deficiencies in Ku80 or Xrcc4 abolish error-free events, showing that accurate end-joining events result from C-NHEJ. The remaining end-joining events (i.e., A-EJ) correspond to deletions at the junctions, with the frequent use of microhomologies distant from the DSB site [1], [2], [11]. Mutagenic events exhibit a similar signature in wild-type cells, suggesting that they result from A-EJ and, thus, that C-NHEJ is not responsible for error-prone DSB repair.

With non–fully complementary ends, end-joining cannot restore a cleavable I-*Sce*I site; therefore, this substrate monitors a single cleavage/joining event. Interestingly, 90–95% of the end-joining events involve 3′-protruding nucleotides that are generated by I-*Sce*I cleavage (Figure 2D.2) [2]. Strikingly, although the four 3′-protruding nucleotides at each end are not complementary, the annealing of two out of the four 3′-protruding nucleotides is observed, corresponding to the maximum possible complementarities. Thus, C-NHEJ adapts to imperfectly complementary ends with minimal genetic modifications. A systematic *in vitro* analysis of most of the DNA end possibilities in human cell extracts consistently yielded similar results [6]. Importantly, in Ku80- or Xrcc4-deficient mammalian cells, the repair events involve none of the 3′-protruding nucleotides, all the resulting products exhibiting deletions at the repair junctions with the frequent use of microhomologies that are distant from the DSB.

These data can be summarized as follows: regardless of the structure of the DNA ends (fully complementary or not), A-EJ removes at least all of the 3′-protruding nucleotides (and generally more), whereas C-NHEJ retains at least one of the 3′-protruding nucleotides, therefore accounting for 90–95% of the events using the 3′-protruding ends.

Importantly, these analyses have revealed that there are two different types of microhomologies (MHs): 1) MHs at the DSB itself that guide the annealing process of imperfectly complementary ends; end-joining is then processed by C-NHEJ in a conservative manner; and 2) MHs distant from the break that are involved in A-EJ, generating extended deletions at the repair junctions (Figure 1B).

These combined data show that C-NHEJ is not error-prone *per se* but is rather versatile and capable of adapting to non–fully complementary ends, maximizing the annealing process of potentially complementary nucleotides, which, in turn, limits genetic alterations. Thus, at the repair junction, C-NHEJ is conservative and the precision of end-joining is dictated by the structure of the DNA ends.

In *Saccharomyces cerevisiae*, sequence analysis of the end-joining events on transfected linearized plasmids revealed that NHEJ is very accurate. In contrast, extended deletions are recovered in *yku70* mutant strains [19], [56], [57]. An alternative end-joining pathway (MMEJ), which increases upon Ku loss, has also been described in a chromosomal context [7]. In addition, NHEJ can generate reciprocal translocations, but in the absence of yKu80, the breakpoint junctions are associated with deletions [58]. An alternative end-joining pathway has also been identified in fission yeast [59].

The continuous expression of endonucleases, such as HO or I-*Sce*I, consistently leads to multiple cycles of cleavage/repair in an essential chromosome, resulting in only 0.1% survival. This result suggests that NHEJ is at least 99.9% error-free because it restores a re-cleavable site [60]–[62]. NHEJ is also adaptable in *S. cerevisiae*. Indeed, the large majority of ends generated by HO are repaired by events involving the four 3′-protruding nucleotides, and the ligation of imperfect overhangs acts in a Ku-dependent manner [62], [63]. Finally, Tdp1, a yeast DNA 3′-phosphatase, has been proposed to increase the accuracy of the NHEJ machinery by preventing the modification of DNA ends [64].

C-NHEJ and A-EJ have also been described in bacteria [65]. In *Mycobacterium smegmatis*, the vast majority of Ku-independent junctions harbor microhomology-mediated deletions, indicating that A-EJ substituted for C-NHEJ during DSB repair [66]. Ku and ligase D are absent in the classical bacterial model *Escherichia coli*, and A-EJ is the most active end-joining pathway in this species [67]. Finally, evidence for conservative C-NHEJ and mutagenic A-EJ pathways has also been presented in plants [12], [68]–[70].

A large number of class II transposons transpose through a cut-and-paste mechanism in which the transposon is excised from its donor site and integrates into another locus where a target site duplication (TSD) is generated on both sides of the newly integrated element. Transposon excision leaves a DSB at the donor site with one copy of the initial TSD at each broken end; DSB repair through end-joining generally yields a characteristic footprint in which the two TSDs are separated by a few bp from the transposon (reviewed in [71]). The excision of cut-and-paste transposons, such as *Sleeping Beauty* [72], [73], *Mos1* [74], or the *P* element [75], has been used in different hosts to induce DSBs at defined genomic loci. These studies have revealed that, in the absence of Ku, large deletions of the flanking sequences are recovered at transposon excision sites. This result confirms that Ku-dependent C-NHEJ is a conservative but versatile repair pathway in mammals, *C. elegans*, and, to a certain extent, *Drosophila*. In the latter, however, a chromosomal assay indicated that the most active end-joining pathway is independent of ligase IV [35].

#### End-joining requiring DNA end processing: The importance of being versatile

**Figure 3:**
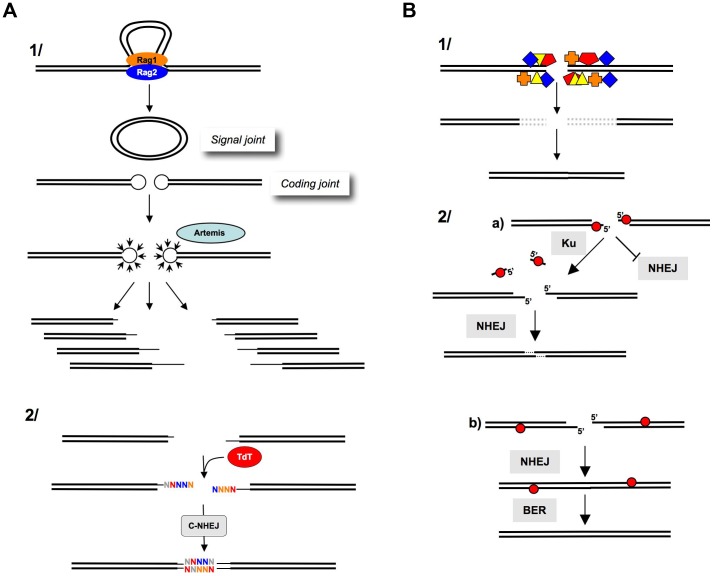
Processing of DNA ends prior to ligation. **A**) **Junctional diversity through V(D)J recombination.** **1**) The Rag1-Rag2 proteins join the V(D)J recombination sites (synapsis step). The cleavage by Rag1-Rag2 generates a circular signal joint and a linear coding joint (containing the coding sequence); however, the cleavage generates hairpins at the extremities, which cannot be directly ligated. The opening of the hairpins (by Artemis) generates a combination of different DNA ends (thin lines), thereby, creating the first level of junctional diversity. **2**) TdT subsequently adds N-nucleotides at the 3′ or blunt ends, creating a second level of junctional diversity. **B**) **After IR.** **1**) IR generates multiple damages at DNA ends (colored boxes). These altered DNA ends are not compatible for enzymatic ligation by ligase IV. The excision of the damaged structures (dotted lines) results in nucleotide deletion after ligation. **2**) **Role of Ku at the DNA ends.** **a**) Abasic sites (red circles) at the DSB inhibit NHEJ. Ku removes these sites, allowing the NHEJ of the processed DNA ends. This reaction results in a limited deletion (one to three nt at the resealed junction). **b**) Abasic sites (red circles) that are far from the DSB do not impair NHEJ. BER can then repair the abasic sites on the resealed molecule. Note that the reduced activity of Ku on these substrates prevents long deletions (from the abasic site to the end), which would result in large deletions at the resealed junction and would avoid the generation of new breaks in the resealed molecule (adapted from [97]).

An efficient immune response absolutely requires genetic diversity at the immunoglobulin gene locus. The first level of diversity is generated through the rearrangement of the (V), (D), and (J) segments induced by the lymphoid-specific Rag1 and Rag2 proteins associated with the ubiquitous C-NHEJ machinery [76]–[79]. V(D)J recombination generates the coding and reciprocal signal joints (Figure 3A), and two steps increase the diversity at the coding joints. First, Rag1/Rag2-mediated cleavage produces hairpins at the broken coding ends (not on the signal ends), and hairpin resolution generates a combination of different sequences at the ends. Second, the addition of N (non-templated) nucleotides by the terminal deoxynucleotidyl transferase (TdT) adds junctional diversity to the coding joints [80]–[82]. Note that the diversity is not generated through C-NHEJ itself but rather through accessory mechanisms (i.e., via a hairpin resolution and TdT). The requirement for additional processes to generate diversity supports the notion that C-NHEJ is not, *per se*, sufficiently mutagenic at the coding joints. Moreover, the repair of signal joints, which results from the direct ligation of blunt ends, is largely error-free [76], [77]. This result shows that when the DNA ends are directly suitable for ligation, C-NHEJ is error-free.

An end-joining process strictly restricted to fully complementary ends would be unable to ligate the coding joints. In contrast, a versatile but conservative process, such as C-NHEJ, is able to join these DNA ends and generate a highly diverse immune repertoire while protecting against side genomic instability. Notably, DNA ends are not complementary during class switch recombination, and the versatility of C-NHEJ is, therefore, essential to complete this process.

IR generates DSBs with chemically altered ends bearing complex lesions that are inept for enzymatic ligation. This situation is different from that of imperfectly complementary ends because the ligase is inactive on those types of chemically modified ends. Thus, IR-induced DSBs must be processed prior to ligation (Figure 3B). Consequently, mutagenesis at the resealed junctions of IR-induced DSBs results from this preliminary “cleaning” step rather than from C-NHEJ itself. Remarkably, Ku possesses a 5′-dRP/AP lyase activity, specialized for DSB, that restricts nucleotide loss at the ends (Figure 3B), therefore maintaining genomic stability [83].

The “cleaning” of IR-induced DSBs generates non-complementary ends. Thus, a non-versatile repair process would be unable to repair IR-induced DSBs, and the organism would be highly sensitive to IR, even at low doses. Therefore, the adaptability of C-NHEJ is essential for resistance to IR. This adaptability should have important consequences for the response to endogenous DSBs and to low exogenous doses, such as environmental or medical (radiological examination) exposures.

### Genome Manipulation: Targeted Mutagenesis Induced by DSBs

The versatility of C-NHEJ has promising applications. Several strategies for targeting mutagenesis are based on mutagenic DSB end-joining. For example, targeted DSBs are generated through different types of nucleases, and unfaithful end-joining events are selected. One could argue that this strategy is primarily based on A-EJ–mediated events and that: i) A-EJ is accompanied by uncontrolled resection at the repaired junctions; ii) A-EJ favors translocations; and iii) in wild-type cells, A-EJ is less efficient than C-NHEJ [1], [2]. For these reasons, selecting strategies that act through C-NHEJ–dependent pathways should minimize the risks of side genomic instability, provided that controlled variability is introduced at the junction. Interestingly, ectopic expression of TdT efficiently adds a limited number of nucleotides at I-*Sce*I-generated ends in a Ku- and ligase IV (C-NHEJ) –dependent manner, also in non-lymphoid cells [84]. One limitation is that TdT preferentially acts on 3′ overhangs or blunt ends. Consequently, TdT should be used in combination with nucleases that generate these types of ends. DNA end–modifying enzymes have also been shown to generate mutations at the resealed junctions of DNA ends generated by TAL endonucleases, but it is unknown whether they act through the C-NHEJ pathway [85].

## Conclusion

C-NHEJ is a conservative end-joining process but permits controlled genetic variability required in essential physiological processes.

At the chromosome level, C-NHEJ protects against DSB movements and profound genome rearrangement. Note that C-NHEJ is involved in physiological processes leading to rearrangements, such as the development of the *Paramecium* macronucleus and V(D)J or class switch recombination. These processes are highly controlled, and the synapsis of the interacting DNA is frequently promoted by associated proteins but not the NHEJ machinery itself, as exemplified in the V(D)J recombination during which the Rag1-Rag2 proteins promote the synapsis of the distant interacting sequences before DNA cleavage. Thus, although C-NHEJ protects against chromosomal rearrangements, it should allow genetic diversity in highly controlled physiological processes.

At the junction sequence level, the previously proposed fallibility of the NHEJ pathway reflects a combination of factors: i) the involvement of the highly mutagenic A-EJ process, ii) the necessity of processing DNA ends prior to their joining, and iii) the versatility/adaptability of C-NHEJ. C-NHEJ is not intrinsically inaccurate, but is versatile and adaptable to imperfect ends, and the actual quality of the end-joining is dictated by the structure of the DNA ends rather than by the C-NHEJ machinery. Versatility/adaptability is paramount for certain essential processes and confers a key role for C-NHEJ in the balance between genetic stability and genetic diversity during the generation of the immune repertoire, molecular evolution, and when challenged with endogenous and environmental sources of DSBs.
